# The effect of written standardized feedback on the structure and quality of surgical lectures: A prospective cohort study

**DOI:** 10.1186/s12909-016-0806-y

**Published:** 2016-11-14

**Authors:** Jasmina Sterz, Sebastian H. Höfer, Bernd Bender, Maren Janko, Farzin Adili, Miriam Ruesseler

**Affiliations:** 1 Department of Surgery, University Hospital Frankfurt, Theodor-Stern-Kai 7, 60590 Frankfurt, Germany; 2 Department of Vascular Surgery, Klinikum Darmstadt, Grafenstraße 9, 64283 Darmstadt, Germany; 3 Department of Trauma, Hand and Reconstructive Surgery, University Hospital Frankfurt, Theodor-Stern-Kai 7, 60590 Frankfurt, Germany

**Keywords:** Lecture, Feedback, Surgery, Peer-feedback, Evaluation, Undergraduate training

## Abstract

**Background:**

Lectures remain an important teaching method to present and structure knowledge to many students concurrently. Adequate measures are necessary to maintain the quality of the lectures. The aim of this study was to determine the impact on the lecture quality using written structured feedback and to compare the ratings of surgical lectures between students and surgical peers.

**Methods:**

Prospective analysis of two consecutive surgical lecture series for undergraduate students at Goethe-University Medical School was performed before and after evaluation of the lecturers via independent written feedback from trained undergraduate students and surgeons. The 22-item feedback instrument covered three areas of performance: content, visualization, and delivery. Additional suggestions for improvement were provided from both students and surgical peers who anonymously attended the lectures. The lecturers, experienced surgeons, as well as the student and peer raters were blinded in terms of the aim and content of the study. Their response to the feedback was collected using a web-based 13-item questionnaire.

The Kendall’s-W coefficient was computed to calculate inter-rater reliability (IRR). Differences between ratings before and after feedback were analyzed using Student’s *t*-test for dependent samples. The Kolmogorov-Smirnov-test was used for independent samples.

**Results:**

A total of 22 lectures from a possible 32 given by 13 lecturers were included and analyzed by at least three surgeons and two students. There were significant improvements in overall score as well as in the details of 9 of the 13 items were found. The average inter-rater reliability was 0.71. There were no differences in the ratings as a function of the rater’s level of expertise (peers vs. students).

We found that 13/23 lecturers (56.5%) answered the questionnaire, and 92% strongly agreed that the written feedback was useful. 76.9% of the lecturers revised their lecture based on the written feedback requiring on average 112.5 min (range from 20 to 300 min).

**Conclusions:**

Overall, this study indicates that structured written feedback provided by trained peers and students that is subsequently discussed by the lecturers concerned is a highly effective and efficient method to improve aspects of lecturing. We anticipate that structured written feedback by trained students that is discussed by the lecturers concerned will improve lecturing.

**Electronic supplementary material:**

The online version of this article (doi:10.1186/s12909-016-0806-y) contains supplementary material, which is available to authorized users.

## Background

Often criticized by teachers and students, lectures are still meaningful in medical education and are an efficient method of teaching [1–4]. Lectures are even more important with high student numbers because the lecturer can present information to many students at the same time and with the same learning outcome regardless of the number of students [5]. Furthermore students can learn from the lecturer’s experience and can discuss academic issues with her or him, and the lecturer can help them to prepare themselves for the faculty’s exams [1]. Medical knowledge continuously increases and is now available from everywhere via the internet. Thus, lectures can help the students to structure, assess and synchronize the available information based on the lecturers’ priorities. However, lectures can definitely be boring or demotivating if badly prepared. Thus, many authors analyze the quality criteria of successful lecturing [1, 5–10] including:

- Appropriate amount of data [5, 6]
- Clearly defined content [6] with clearly stated goals of the talk [9]
- Interactions with the audience [5, 9–11]
- Coherent and well prepared slides [5, 9]

To improve one’s own teaching quality - especially regarding lecturing - a critical self-reflection of one’s own performance is necessary. Here, feedback plays an important role, because it improves knowledge and competence and helps to reflect on one’s performance [12–15].

However, non-specific, unclear, and irrelevant feedback is useless and may hinder the learning process [13].

Thus, feedback must fulfill defined quality criteria to be successful [12, 16, 17]. It must be constructive, specific and offer concrete suggestions for improvement [12, 17]. Furthermore it should be based on direct observations and be made timely [17, 18].

The easiest way to give feedback is orally. Direct talk between the person offering feedback and the receiver can be initialized via oral feedback. This feedback should be timely because important aspects might be forgotten if delayed due to the spontaneous character of oral feedback [19].

Another way of giving feedback is written feedback. It provides an enduring record and reference point that can be taken home [19]. This allows the receiver (e.g. the lecturer) to reflect on it repetitiously and to reread the personal feedback while they revise their lectures [11]. It also allows the receivers to directly compare with others [19]. Haghani et al. demonstrated that written feedback had a higher learning effect than oral feedback [20]. However, written feedback requires a time consuming preparation [19].

Feedback on teaching quality by peers has been shown to be very successful in terms of improving teaching quality. It is highly accepted by feedback providers and recipients [21–28].

Despite the advantages of peer feedback, students must realize the structure, learning objectives and aims of the lectures. The content and context must be presented more clearly to students than experts. Thus, their feedback is critical. On the other hand, student’s evaluations are influenced by many factors that cannot be influenced by the lecturer and lecture’s quality including age [29], expected grades in the relating tests [30], intrinsic motivation or general interest in the topic [31]. In fact, the lecture rating by students is often influenced by how entertaining the lecture is. Thus, a combination of student and peer feedback might be reasonable.

The aim of this study is to analyze if student and expert raters are using the feedback sheet differently and to measure the impact of written, structured feedback on the quality of a lecture series in surgery for undergraduate medical students. We also want to analyze the lecturers’ response to this kind of feedback.

## Methods

### Study design

This study has a prospective design and analyzed the effect of structured written feedback given by students and peers on the lecture quality of a surgical lecture series for undergraduate medical students. The study was conducted according to ethical principles of the Helsinki Declaration (Ethical Principles for Medical Research Involving Human Subjects) and was approved by the ethics committee of the medical faculty of the Goethe University, Frankfurt, Germany.

### Study protocol

The lecture series is part of the surgical curriculum for undergraduate medical students at Frankfurt Medical Faculty [32]. It takes place twice a year over an 8-week period for 4th year students in a six-year program. It consists of 32 lectures with a duration of 90 min each. The lectures cover the main topics in surgery as defined in the catalogue of learning objectives from the German Society of Surgery [33]. Table 1 shows the distribution of surgical disciplines.

**Table 1:** Epidemiological data of the study participants

	Lecturer	Expert reviewer
Number	13	4
Age (Years)	51.8 ± 5	30.5 + 3.7
Gender (m/f)	All male	1 male, 3 female
Rank (*n*)		
Resident	0	3
Consultant	1	1
PhD/Assistant Professor	2	0
Professor	10	0
Discipline (*n*)		
General Surgery	2 (3 lectures included)	1
Vascular Surgery	1 (3 lectures included)	0
Cardiothoracic Surgery	4 (5 lectures included)	0
Pediatric Surgery	1 (3 lectures included)	0
Cranio-Maxillofacial and Facial Surgery (CMF)	1 (1 lecture included)	1
Trauma Surgery	4 (7 lectures included)	2

The students’ attendance of the lectures is optional. However, the lecture series ends with an obligatory 50 item multiple-choice examination. Passing the examination is a prerequisite for participating in the following courses in the curriculum.

The lectures are given by experienced surgeons from the University hospital. They participate in undergraduate surgical training as part of their role as a medical teacher. Data were obtained from all lecturers regarding age and years of lecturing experience. The evaluation of all courses is a mandatory component based on the national regulations to study medicine. Still, lecturers were informed over the particular evaluation which was performed in all surgical lectures and had the possibility to dissent the use of their evaluation data for study purpose. Lecturers were blinded regarding the contents of the evaluation process and aim of the study.

### Measurement

The study took place from April to June 2014 (lecture series 1) and October to December 2014 (lecture series 2). The evaluation sheet used was described by Ruesseler et al. [25] and is based on the publications by Newman [34, 35] as well as the quality criteria for lectures published in the literature [1, 5, 6]. Additional file 1 The 22- item instrument was divided into three categories: content/structure (10 items), visualization (5 items) and presentation (7 items). Each item was rated on a 5-point Likert scale (from 1 = did not show to 5 = excellent) with descriptive benchmarks for excellent (5), adequate (3) and poor performance (1) for each item [25, 34, 35]. Furthermore, each rater had to document the timetable of each lecture and describe the strength of each lecture and give suggestions for improvement.

The reviewer team consisted of 4 surgeons (peers) and 3 undergraduate medical students. The students attended the lecture series regularly; however, they received a compensation of 10 € per hour for participating as a reviewer. The reviewers were blinded for the study aim. To increase the inter-rater reliability, all reviewers received a two-hour training prior to the first and the second lecture series [25, 34]. During this training, they rated a videotaped lecture using the evaluation sheet. Afterwards, they discussed definitions, items, and their results with each other and talked about common rater errors (e.g. halo-effect).

Each of the 32 lectures was evaluated by at least two raters—one student and one surgeon (peer). They rated the lecture simultaneously and independently without agreement. Both lecture series were rated similarly. All student raters and most peers changed after the first lecture series to minimize the rerun bias.

All lecturers received standardized written feedback on their lecturing performance for each lecture three weeks prior to the second lecture series. For this, all ratings of a single lecture were averaged to a single score for each item on the assessment sheet. In their written feedback, each lecturer received the mean score of all lecturers for each item, the best and the worst score of all lecturers, as well as his own averaged score for each item. Furthermore, they received the timetable of their lecture and their lecture’s strengths and suggestions for improvement—especially for poorly scored items.

The results were anonymously presented and discussed during the monthly meeting of surgical medical teachers. The second lecture series was assessed and analyzed as described above. The lecturers’ reaction to the feedback was collected using a web-based questionnaire consisting of 13 items. The questionnaire was sent to all lecturers via email after their second lecture and prior to receiving their second feedback. A reminder to take the survey was sent after two weeks.

The dataset supporting the conclusions of this article and the evaluation sheet is included within the article and its additional files.

### Statistical analysis

Statistical analysis used Microsoft Excel (Microsoft Corporation, Redmond, USA) for the personal characteristics of raters and lecturers and evaluation. SPSS Statistics version 19 (IBM, Armonk, USA) was used for the checklist results. After verifying the Gaussian distribution of the data, the values were presented as the mean ± standard deviation. The Kendall’s W coefficient was computed to calculate the inter-rater reliability (IRR). The rating differences before and after the feedback were analyzed using Student’s *t*-test for dependent samples as well as the Wilcoxon signed-rank test. For independent samples, we used the Student’s *t*-test for independent samples and the Kolmogorov-Smirnov test for independent samples.

## Results

Each lecture series consisted of 32 lectures. For some lecture topics, the lecturer changed between series 1 and 2. These lectures were excluded from the study. Thus, a total of 22 lectures by 13 lecturers were included and analyzed. The epidemiological data of the lecturers are presented in Table 1.

Four surgeons (peers) (3 in series 1 and 4 in Series 2) and 3 undergraduate medical students (1 in series 1 and 2 in Series 2) were part of the reviewer team. The epidemiological data of the surgeons are presented in Table 1. The lectures were evaluated by 1.22 expert raters on average (maximum 3, minimum 0) and by 1.13 student raters on average (maximum 2, minimum 0).

### Impact on content, structure and quality of the lectures

**Fig. 1:**
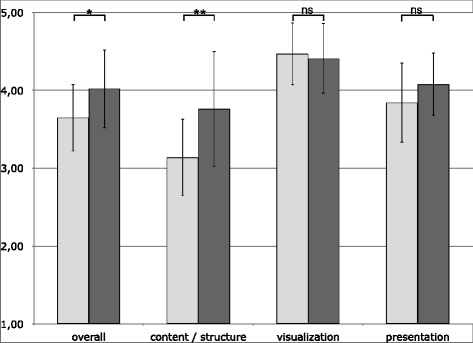
Mean rating of the evaluation in series 1 and series 2. Presented as mean ± std. dev. for the overall score and the main categories. Each item was rated on a 5-point Likert scale (from 5 = excellent to 1 = poor). In light grey ‘point of measurement 1’; in dark grey ‘point of measurement 2’; * *p* < 0.05; ** *p* < 0.01; ns *p* > 0.05

Figure 1 shows the overall results for series 1 and series 2 as well as the results for the three main categories. Significant improvements were found in the overall score and in the category ‘content/structure’. In the category ‘presentation’, the results tended to improve, but no significant changes were found. In the category ‘visualization’, the lecturers achieved good results similar to series 1. In this category, the results remained high without significant changes.

The detailed results for each item are presented in Table 2. For 9 items, we found significant differences in the scores after written feedback. Most of these were in the category ‘content/structure’. In the ‘presentation’ category, we found significant changes in two items. No significant changes were found in the category ‘visualization’.

**Table 2:** Comparison of the results of the evaluation of all items for series 1 and 2

Item	Series 1	Series 2	*p*
Content/Structure			
Clear and organized presentation	3.58 ± 0.7	4.05 ± 1.1	**0.038**
Presenting an advanced organizer	2.97 ± 0.86	3.7 ± 1.46	0.091
Presents goal of the talk	1.86 ± 1.00	3.01 ± 1.83	**0.012**
Key concept	3.89 ± 0.82	3.62 ± 1.14	0.44
Audience interaction	3.58 ± 0.85	4.03 ± 0.6	**0.047**
Appropriate amount of data	4.23 ± 1.02	4.49 ± 0.56	0.337
Linking to previous knowledge	3.11 ± 0.7	3.98 ± 0.72	**< 0.001**
Clear algorithm	3.22 ± 0.86	3.8 ± 0.92	**0.009**
Conclusion	1.7 ± 1.07	3.05 ± 1.53	**0.002**
Time management	2.89 ± 1.51	4.0 ± 0.72	**0.002**
Visualization			
Appropriate number of slides	4.5 ± 0.9	4.55 ± 0.42	0.740
Adequate slide design	4.61 ± 0.53	4.34 ± 0.79	0.087
Adequate audio and visual aids	4.45 ± 0.63	4.46 ± 0.55	0.743
Adequate amount of text	4.26 ± 0.52	4.11 ± 0.75	0.448
Congruence of text and visual aids	4.51 ± 0.64	4.61 ± 0.52	0.376
Presentation			
Speech flow	4.07 ± 0.94	4.47 ± 0.57	**0.049**
Audibility and pronunciation	4.08 ± 0.74	4.36 ± 0.67	0.214
Enthusiasm for the topic	3.63 ± 0.75	3.95 ± 0.74	0.072
Respect for the audience	3.29 ± 0.7	3.43 ± 0.55	0.326
Invitation to questions	3.67 ± 0.76	4.03 ± 0.74	**0.036**
Clear sequence and development of the talk	3.85 + 0.65	4.16 + 0.89	0.325
Language of slides	4.25 + 1.1	4.29 + 0.72	0.977

Results per item on average for series 1 and series 2, presented in mean ± std. dev. Each item was rated on a 5-point Likert scale (from 5 = excellent to 1 = poor)

*p*<.05 was rated as significant (bold)

There were no significant changes in the average length of the lectures from the first to the second lecture series (86.43 min ±5.78 in series 1 and 85.78 min ±11.21in series 2). The average length was 85 min for both series.

### Inter-rater reliability

To determine the IRR of the lecture series evaluation, the results of all lectures were used (independent of study inclusion of the lecturer). The IRR (Kendall W) was 0.70 ± 0.12 on averaged for all reviewed lectures and 0.71 ± 0.11 for the included lectures. It was above 0.6 in 81% of the lectures. In 19%, it was between 0.4 and 0.6. For lecture series 1, the IRR was above 0.6 in 28 of the 29 lectures. It was between 0.4 and 0.6 in 1 of 29 lectures. In lecture 2, the IRR was above 0.6 for 20 of the 30 lectures and between 0.4 and 0.6 for 10 of the 30 lectures. None of the IRR values were below 0.4.

There were no differences in the ratings as a function of the rater’s level of expertise (peers vs. students) (Table 3).

**Table 3:** Correlation between level of expertise and ratings

	Correlation coefficient Kendall-Tau-b	*p*
Overall	0.190	0.002
Content/Structure	0.167	0.009
Visualization	0.035	0.600
Presentation	0.228	< 0.001

### Results of the questionnaire

**Fig. 2:**
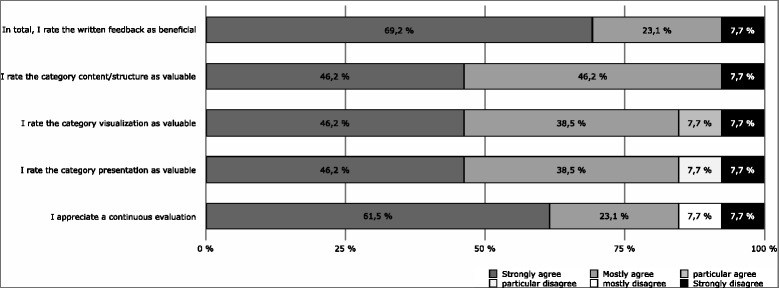
Results of lecturers’ questionnaires. Presented as a 6-point-Likert-Scale

We found that 13 of the 23 lecturers (56.5%) answered the questionnaire. Whilst more than 92% strongly agreed or agreed with the statement ‘In total, I rate the written feedback as beneficial’, only one lecturer disagreed (Fig. 2).

76.9% of the lecturers reported that they revised their lecture based on the written feedback requiring on average 112.5 min (range from 20 min to 300 min). They stated that the precise recommendations for improvement were extremely useful.

## Discussion

Feedback is essential for learning [12–15, 36]. In this study, we demonstrated the effects of structured written feedback given by students and peers on a lecture series in surgery. The strongest improvement from the written feedback was shown in the category content/structure—especially regarding the items “Presents goal of the talk” or “Providing a clear algorithm”. These are essential for the students as they improve learning aptitude [1].

Ruesseler et al. hypothesized that improvements in the category “Presentation” can only be achieved after didactical training based on the results of their study [25]. In contrast to these findings, we demonstrate improvements in the category “Presentation”, especially for the items “Speech flow” and “Invitation to questions”. We found that these results as well as “Time management” increased significantly. In our opinion, this is one of the main reasons for the increased results—the lecturers simply had more time to take care of the speech flow because of more effective time management. In addition, the lecturers in Ruesseler et al. already achieved superior results in this category by the first lecture series. Thus, it was more difficult to improve here because their results were already good.

Consistent with previous findings [11, 22–24, 37] we demonstrated the high acceptance of feedback given by peers—even if the lecturers were blinded regarding the evaluation process. Most lecturers rated the written feedback as beneficial and revised their lectures after the feedback. Unlike these existing studies, we decided to blind the lecturers under evaluation. Thus, we could measure the unadulterated effect and reaction to the feedback without biasing it by only evaluating those lecturers willing to be evaluated. This could correspond to those who were already motivated to teach effectively. However the generalizability of the questionnaire results is limited because only as 56% of our lecturers responded to the questionnaire.

On the other hand, the risk of negative reactions to unheralded feedback is much higher. Consistent with the findings of Eraut et al. [12], we were confronted with some misinterpretations of the intended feedback - especially for “Presentation”, e.g., one lecturer misunderstood some items as an estimation of his own personal behavior. To minimize these misapprehensions, many authors describe pre-observation meetings [22, 23, 38, 39]. Even if we decided not to hold these meetings because of the reasons described above, we will proceed with the evaluations of the lectures in this lecture series—future lecturers will be informed about the ongoing evaluation. Thus, based on the lecturer’s evaluation on the past lecture series, the reviewers and lecturers harmonized topics of personal interest via the following evaluation.

Each lecturer received written feedback consisting of his own evaluation embedded within the anonymized results of the best and worst lecturer as a ranking. Following the first feedback and a growing discussion between the lecturers regarding their ratings, we observed a growing competition between the lecturers. That is, “If Prof. X has a case presentation and is well rated, then I’ll do the same!” This competition can highly improve a lecture if the lecturer uses the written standardized feedback. However, it can also be disadvantageous, e.g., one of the lecturers focused only on improving his presentation of learning objectives. The outcome here was a lecture that presented the importance of learning objectives and his learning objectives of the present lecture for about 20 min. This caused him to run out of time for remainder of his lecture, which he did not revise. On the other hand, those lecturers who already achieved good results and who were best ranked in comparison to all other lecturers did not see the necessity to change.

We showed high IRR between all reviewers using the standardized evaluation sheet. However, this might be a limitation because not all reviewers changed after the first lecture series. Thus, these reviewers could be biased by their experiences in the first lecture series. This bias was not seen because of the high IRR between all reviewers—both expert and student groups as well as persisting and interchanged reviewers.

Based on our results, we demonstrated that the results and quality of the evaluation are not influenced by the reviewers’ level of training when using a standardized evaluation sheet as presented here. Our study shows that even student ratings based on a validated evaluation sheet are comparable to evaluations created by peers using the same metric. We confirmed that the bias in student evaluations as emphasized in the literature [29–31] can be minimized using a validated questionnaire and reviewer training. The training does not need to be longer than an hour. This method is an efficient option for a good and valid evaluation/feedback and thus we decided to have further evaluations done only by trained students. This facilitated evaluations with lower personnel costs. In an upcoming project, we will evaluate if the positive impact of this kind of feedback will be persistent when only students provide feedback.

This study is limited in that we did not analyze the effects of the revised lectures on students’ medical knowledge acquisition. To the best of our knowledge, such a study has not yet been conducted yet—perhaps because of the challenges inherent in designing such a study, e.g. designing a test with comparative difficulty and comparative tested learning objectives in two consecutive semesters without the two student groups exchanging the test items. Another limitation is the lack of a control group. Because of the small number of lecturers in the lecture series, we made a conscious decision to not create a control group. Other than these limits, the effectiveness of any type of feedback compared to giving no feedback is already proven. Thus, the control group would have been disadvantaged from the beginning.

In this study, lecturers who received good evaluations asked for a certificate of their teaching performance within this lecture series. They suggested that they needed tribute for being a good teacher from the faculty. This is consistent with the results of Müller-Hilke [40]. She demonstrated that medical teachers’ highest motivation is “Fame and glory”. To boost the tribute to excellent teaching, the educational committee of the department of surgery at our faculty decided to perpetuate the evaluation and to implement an award for the most highly evaluated lecturer as well as the best evaluated department.

## Conclusions

Overall, this study indicates that structured written feedback provided by trained peers and students and discussed by the lecturers concerned is a highly effective and efficient method to improve aspects of lecturing. We anticipate that structured written feedback by trained students and discussed by the lecturers concerned will improve lecturing.

## Additional file

Additional file 1: Evaluation sheet lecturers in surgery. (DOCX 98 kb)

## Abbreviations

IRR: Inter-rater reliability
