# Supplementary material for: The effect of written standardized feedback on the structure and quality of surgical lectures: A prospective cohort study
Source: BMC Med Educ. 2016 Nov 14;16:292. doi: 10.1186/s12909-016-0806-y (PMC5109746; doi:10.1186/s12909-016-0806-y)
Supplement: Additional file 1: — Evaluation sheet lecturers in surgery. (DOCX 98 kb) [file 12909_2016_806_MOESM1_ESM.docx]

**Date:** ____________ **Duration:** _________ min **Reviewer**: _______________________

**Lecturer:** ________________________________  **Topic:** ________________________

**Begin as scheduled**: _________ **Actual beginning**: _________________________

**Reasons for delay**: ______________________________________________

| **Duration** | **Main topics** | **Comments** |
| --- | --- | --- |
|  |  |  |
|  |  |  |
|  |  |  |
|  |  |  |
|  |  |  |
|  |  |  |
|  |  |  |
|  |  |  |
|  |  |  |
|  |  |  |
|  |  |  |
|  |  |  |
|  |  |  |
|  |  |  |
|  |  |  |
|  |  |  |
|  |  |  |
|  |  |  |
|  |  |  |
|  |  |  |
|  |  |  |
|  |  |  |
|  |  |  |
|  |  |  |
|  |  |  |
|  |  |  |
|  |  |  |
|  |  |  |
|  |  |  |
|  |  |  |
|  |  |  |
|  |  |  |
|  |  |  |
|  |  |  |
|  |  |  |
|  |  |  |
|  |  |  |
|  |  |  |
|  |  |  |
|  |  |  |

| **Content and Structure** | | | | | |
| --- | --- | --- | --- | --- | --- |
|  | **5**  *Excellent demonstration of performance* | **4** | **3**  *Adequate demonstration of performance* | **2** | **1**  *Does not demonstrate* |
| Presents material in a clear, organized fashion | Uses an explicit, organized framework so that the presentation flows logically (e.g. articulates a structure and sequence to the talk, frames subtopics, links concepts) |  | Presentation has some organization, but limited in structure, linkage, and/or sequence. |  | Does not present material in a clear, organized fashion. |
| Provides a brief outline of the goals of the lecture | During introduction, communicates purpose of the presentation. For example, may provide an overview of content, explains relevance of topics |  | States the goals, but  description is limited in scope (e.g. *only* states topics to be covered or provides the format of talk). |  | Does not provide overview or communicate the goals of talk. |
| Clearly states goals of the talk | During introduction, states expected learning outcomes in detail |  | States some learning objectives of the talk, but not structured and not detailed and defined |  | Does not present the learning objectives of the talk |
| Explains and summarizes key concepts | Defines new terms/principles, synthesizes information (e.g. identifies important points; uses examples, analogies, metaphors; thinks out loud). |  | Explains some key concepts, or provides vague explanations. |  | Does not explain or summarize key concepts. |
| Actively involving the student - encourages appropriate audience interaction | Stimulates active participation (e.g. makes eye contact, solicits comments and questions, polls the audience, uses deliberate silence, poses open-ended questions, invites learners to interact with each other; manages flow of discussion). |  | Encourages some interaction or uses less effective strategies (e.g. asks ‘closed’ questions, offers little wait time, often turns back to audience, and reads from slides). |  | Does not engage or encourage interaction (e.g. reads all slides without looking at audience; defers questions, yet does not answer them). |
| Appropriate, limited data | The amount of data presented is appropriate for the talk. |  | The presentation contains in some parts too much information for the time of the lecture |  | The lecture in total is too crowded regarding the amount of data presented |
| Successful linking with previous knowledge | The lecturer links his talk to students’ previous knowledge, e.g. by posing questions at the beginning of the talk |  | The linking to previous knowledge is only performed to a limited extent |  | There is no linking to previous knowledge |
| Provides a clear algorithm | A clear algorithm regarding approach to the presented emergency is provided |  | The algorithm is only presented in some parts |  | No algorithm is presented. |
| Provides a conclusion to the talk | Concludes presentation by synthesizing information, summarizing main points, and inviting/responding to questions (e.g. repeats or rephrases questions as needed). Open to hearing learners’ perspectives and opinions |  | Provides synthesis and/or summary of talk, but this is limited in scope. Invites few questions and/or provides limited or ambiguous responses. |  | Does not synthesize or summarize information. |
| Time management | The timing is adequate, thus there is no rushing at the end of the talk, there is sufficient time for conclusion, take home messages and questions/discussion. |  | Due to some mismanagement in timing, there is some rush through the lecture. However, there is some time for conclusion and questions |  | Due to time constraints, there is no time for conclusion and questions. |
| **Visualization** | | | | | |
|  | **5**  *Excellent demonstration of performance* | **4** | **3**  *Adequate demonstration of performance* | **2** | **1**  *Does not demonstrate* |
| Appropriate number of slides | Number of slides is appropriate for length of lecture (Average: max. 2 slides per lecture minute) |  | Number of slides is to some extent excessive, causing crowding |  | There are far too many slides for the length of the lecture. |
| Adequate design (text, pictures, colours, fond) | The design of the slides is adequate regarding text size, pictures and colour (good visibility and legibility, adequate size of text) |  | In some slides visibility and legibility is limited due to the slide design |  | Slide design is not appropriate, slides are not adequate visible and legible. |
| Clear graphics/scheme, appropriate animation | Sensitive to the setting and tailors audio and visual aids so all can see and hear (e.g., checks if audience can hear/see material; talks to audience not to blackboard, laptop, or screen; visual material is well organized, text is legible, and graphics are clear). |  | At times audiovisuals are inaudible/illegible. |  | Audiovisuals  are often inaudible/ illegible. |
| Simplicity (key words, short sentences), amount of words | The text on each slide is in ‘catchword’ form or only very short sentences. There is a maximum of 5x7 words per slide |  | On some slides, the number of words is excessive or they contain some long sentences. |  | Most slides are overcrowded with complex sentences and too many words. |
| Audio and/or visual aids  reinforce the content effectively | Appropriately chooses and designs instructional material to emphasize key points, demonstrate relevance of material, or stimulate thought. |  | Some of the audio and/or visual aids reinforce content, or material is less than effective (e.g. slides are cluttered) |  | Audio and/or visual aids do not reinforce content. |
| **Presentation** | | | | | |
|  | **5**  *Excellent demonstration of performance* | **4** | **3**  *Adequate demonstration of performance* | **2** | **1**  *Does not demonstrate* |
| Speech flow and pauses | Speech flow and pausing are appropriate. |  | At some times, speech flow is too fast or too slow. |  | Speech flow is far too slow or fast without pausing or with inappropriate pausing. |
| Voice and articulation | Voice and pronunciation are clear |  | At times, voice is unclear |  | Voice is unclear |
| Shows enthusiasm for the topic | Demonstrates keen enthusiasm for topic through voice, eye contact, energy, movement and/or body language (e.g. varies pitch, inflection, tempo, and volume; gestures to emphasize importance). |  | Shows some enthusiasm for topic, but limited in display. |  | Does not show  enthusiasm for the topic. |
| Respect and politeness towards the audience | Lecturer demonstrates excellent respect for the audience by addressing the audience, politeness |  | Adequate |  | No |
| Invitation for questions, comments, objections | Lecturer invites questions and comments to the talk and provokes objections |  | Lecturer sometimes invites questions and comments |  | Lecturer does not invite questions. |
| Adequate moderation of the talk | Lecturer presents a clear sequence and development of the talk. |  | In some parts, lecturer presents a clear sequence of the talk |  | There is no clear sequence and development of the talk. |
| Sildes’ language | All slides were in German |  | Some slides were not in German |  | Almost all slides were not in German |

**The following learning goals were covered in the lecture:**

| **Learning goal** | Level of expertise | | Clinical diagnostics | | Apparatus-based diagnostics | | Therapy | | Emergency | | Management | | **Comments** |
| --- | --- | --- | --- | --- | --- | --- | --- | --- | --- | --- | --- | --- | --- |
|  | C | L | C | L | C | L | C | L | C | L | C | L |  |
|  |  |  |  |  |  |  |  |  |  |  |  |  |  |
|  |  |  |  |  |  |  |  |  |  |  |  |  |  |
|  |  |  |  |  |  |  |  |  |  |  |  |  |  |
|  |  |  |  |  |  |  |  |  |  |  |  |  |  |
|  |  |  |  |  |  |  |  |  |  |  |  |  |  |
|  |  |  |  |  |  |  |  |  |  |  |  |  |  |
|  |  |  |  |  |  |  |  |  |  |  |  |  |  |
|  |  |  |  |  |  |  |  |  |  |  |  |  |  |
|  |  |  |  |  |  |  |  |  |  |  |  |  |  |
|  |  |  |  |  |  |  |  |  |  |  |  |  |  |
|  |  |  |  |  |  |  |  |  |  |  |  |  |  |
|  |  |  |  |  |  |  |  |  |  |  |  |  |  |
|  |  |  |  |  |  |  |  |  |  |  |  |  |  |
|  |  |  |  |  |  |  |  |  |  |  |  |  |  |
|  |  |  |  |  |  |  |  |  |  |  |  |  |  |
|  |  |  |  |  |  |  |  |  |  |  |  |  |  |
|  |  |  |  |  |  |  |  |  |  |  |  |  |  |
|  |  |  |  |  |  |  |  |  |  |  |  |  |  |
|  |  |  |  |  |  |  |  |  |  |  |  |  |  |
|  |  |  |  |  |  |  |  |  |  |  |  |  |  |

| **Duration** | **Main topics** | **Comments** |
| --- | --- | --- |
|  |  |  |
|  |  |  |
|  |  |  |
|  |  |  |
|  |  |  |
|  |  |  |
|  |  |  |
|  |  |  |
|  |  |  |
|  |  |  |
|  |  |  |
|  |  |  |
|  |  |  |
|  |  |  |
|  |  |  |
|  |  |  |
